# Genome-wide identification, phylogenetic and expression pattern analysis of *GATA* family genes in *Brassica napus*

**DOI:** 10.1186/s12870-020-02752-2

**Published:** 2020-12-04

**Authors:** Weizhuo Zhu, Yiyi Guo, Yeke Chen, Dezhi Wu, Lixi Jiang

**Affiliations:** Department of Agronomy, Key Laboratory of Crop Germplasm Resource of Zhejiang Province, Zhejiang University, Hangzhou, 310058 China

**Keywords:** *Brassica napus*, GATA, Genome-wide, Expression patterns, SNP distribution

## Abstract

**Background:**

Transcription factors *GATAs* are involved in plant developmental processes and respond to environmental stresses through binding DNA regulatory regions to regulate their downstream genes. However, little information on the *GATA* genes in *Brassica napus* is available. The release of the reference genome of *B. napus* provides a good opportunity to perform a genome-wide characterization of *GATA* family genes in rapeseed.

**Results:**

In this study, 96 *GATA* genes randomly distributing on 19 chromosomes were identified in *B. napus*, which were classified into four subfamilies based on phylogenetic analysis and their domain structures. The amino acids of BnGATAs were obvious divergence among four subfamilies in terms of their GATA domains, structures and motif compositions. Gene duplication and synteny between the genomes of *B. napus* and *A. thaliana* were also analyzed to provide insights into evolutionary characteristics. Moreover, *BnGATAs* showed different expression patterns in various tissues and under diverse abiotic stresses. Single nucleotide polymorphisms (SNPs) distributions of *BnGATAs* in a core collection germplasm are probably associated with functional disparity under environmental stress condition in different genotypes of *B. napus*.

**Conclusion:**

The present study was investigated genomic structures, evolution features, expression patterns and SNP distributions of 96 *BnGATAs.* The results enrich our understanding of the *GATA* genes in rapeseed.

**Supplementary Information:**

The online version contains supplementary material available at 10.1186/s12870-020-02752-2.

## Background

Transcription factors (TFs) regulate gene expression by recognizing and combining *cis*-acting elements on the promoter regions of target genes [1]. TFs play key roles in plant developmental processes, hormones signaling pathways and disease resistance responses. There are several well-known transcription factor families including *WRKY*, *MYB* (*V-myb avian myeloblastosis viral oncogene homolog*), *DREB* (*Dehydration-responsive element-binding protein*), *bZIP* (*Basic region-leucine zipper*), *MADS-box* and *GATA* (GATA-binding factor) in plants. Among them, the *GATA* genes are characterized as important regulators for many biological processes, such as flower development, carbon and nitrogen metabolisms [2]. The *GATA* genes could recognize and bind to the (T/A)GATA(A/G) sequences to regulate the transcription levels of their downstream genes [3, 4]. The DNA binding domains of the GATA proteins contain a type IV zinc finger structure C-X_2_-C-X_17–20_-C-X_2_-C and a conserved basic follow region, and most of them featured with C-X_2_-C-X_18_-C-X_2_-C or C-X_2_-C-X_20_-C-X_2_-C zinc finger domains [2, 3, 5, 6]. Generally, the *GATA* family genes could be divided into four subfamilies as subfamily I, II, III and IV in *Arabidopsis thaliana* based on the phylogenetic relationships, DNA binding domains and intron-exon structures [2, 5, 7–9].

Many studies have been proved that the *GATA* TFs are responsible for plant growth development, flowering, chlorophyll synthesis, greening and senescence. For instance, the loss-of-function and the over-expression of the *GATA* genes such as *GNC* (*GATA*, Nitrate-inducible, Carbon-metabolism) and *GNL* (*GNC*-like) can change flowering time and chlorophyll synthesis in *A. thaliana* [10–13]. *GNC* regulates downstream genes such as the light-labile factors *PIFs* (*phytochrome interacting factors*) to control chloroplast biogenesis and stomatal index [10, 13]. The cross-repressive interactions between *GNC*/*GNL* and *MADS-box* transcription factor *SOC1* (*Suppressor of Overexpression of Constans1*) affect flowering time [12, 13]. Besides, *GNC* and *GNL* are considerable repressors of gibberellin signaling through being regulated by DELLA and *PIF* regulators [10, 14]. Moreover, auxin response factors *ARF2* and *ARF7* can repress the expression of *GNC* and *GNL* genes [10–12, 14]. In *Brassica napus*, a *GATA* member *BnA5.ZML1* was reported to be a stigma compatibility factor [15]. *PdGNC* in *Populus* plays a crucial role in photosynthesis and plant growth [16]. In wheat, over-expression of *TaZIM-A1,* a member of the *GATA* family, caused the delay of flowering and the decrease of thousand-kernel weight [17].

The *GATA* TFs also respond to diverse abiotic stresses in plants. Under cold stress, the expression levels of *GNC* and *GNL* were significantly increased, while the seedling survival ratio was elevated in the over-expression lines with *GNC* or *GNL* genes in *A. thaliana* [18]. Moreover, under low temperature, *GATA9* gene showed remarkably changed expression to activate its downstream genes in *Vigna subterranea* [19]. Under salinity stress, *OsGATA8* overexpressed lines showed higher biomass accumulation and photosynthetic efficiency than the wild-type and the knockdown seedlings of rice [20]. In soybean seedlings, the expression of *GATA44* and *GATA58* genes were extremely down-regulated under low nitrogen settlement [21]. In *B. juncea,* 29 *GATA* genes responded to high temperature and drought treatments by their transcription levels based on the RNA-seq experiments [22].

Rapeseed is an important oil crop. To date, the genomes of Darmor-*bzh* (winter ecotype), Tapitor (winter ecotype), Zhongshuang 11 were successfully sequenced and assembled [23–25]. Recently, we re-sequenced 991 accessions from the global rapeseed germplasm and established a worldwide core collection [26, 27]. In this study, 96 *GATA* genes were identified and characterized in the genome of *B. napus*. Moreover, the expression pattern and SNPs distribution of these genes were analyzed. The main objectives of the present study are to (i) investigate the difference of gene/protein sequences and genetic structures of *BnGATA*s; (ii) determine the gene expression patterns in tissues and under abiotic stresses; and (iii) identify SNPs of *BnGATA*s in a worldwide core collection. These results enrich our knowledge about *BnGATA* genes, providing a basis of molecular characteristics and facilitating breeding marker-assisted breeding in rapeseed.

## Methods

### Identification of *GATAs* in *B. napus*

The amino acid sequences of the GATA family members in *A. thaliana* were obtained according to a previous study (Table S1, [2]), and the homologs of GATAs in *B. napus* were blasted against the reference genome of the rapeseed cultivar “Darmor-*bzh*” (v4.1 genome, http://www.genoscope.cns.fr/brassicanapus/data/). Hidden Markov Model (HMM) and BLASTP programs were applied for the identification of BnGATA proteins. The HMMER profile of GATA zinc finger domain (PF00320) from the Pfam database (http://pfam.janelia.org/) was used to perform the local BLASTP (E-value-20) search. The candidate sequences of GATAs were confirmed in the SMART database (http://smart.embl-heidelberg.de/) [28], the NCBI Conserved Domain database (http://www.ncbi.nlm.nih.gov/Structure/cdd/wrpsb.cgi) [29] and the Pfam database [30]. Subfamily members were named based on their arrangement order on chromosomes of the *B. napus* genome (Table S2). Moreover, the length of amino acids, molecular weights (MW) and isoelectric point (pI) of GATA proteins were calculated using tools from ExPASy (http://www.expasy.ch/tools/pi_tool.html).

### Phylogenetic analysis and classification of *GATAs*

The multiple alignments of GATA amino acids were done using the ClustalW with default parameters [31]. A phylogenetic tree was constructed using the MEGA 7.0 by the Neighbor-Joining (NJ) method [31, 32], with the following parameters: poisson model, pairwise deletion and 1000 bootstrap replications. Unrooted NJ tree of GATA proteins from *A. thaliana* and *B. napus* was also constructed using the MEGA 7.0. The *GATA* family members from *A. thaliana* were referred to classify the *GATA* family members in *B. napus*. In addition, the conserved GATA zinc finger domains in proteins were identified using the MEGA 7.0 and the GeneDoc software.

### Motifs and gene structures

The Gene Structure Display Server online program (GSDS: http://gsds.cbi.pku.edu.ch) was used to analyzed exon-intron structures of all *GATA* genes [33]. To identify conserved motifs in GATA proteins, the Multiple Expectation Maximization for Motif Elicitation (MEME) online program (http://meme.sdsc.edu/meme/itro.html) was performed with the following parameters: number of repetition = any, maximum number of motifs = 10; and optimum motif length = 6 to 100 residues [34].

### Chromosomal localization and gene duplication analyses

The distribution of 96 *GATA* genes identified in *B. napus* was mapped to 19 chromosomes according to their physical location information from the reference genome database (http://www.genoscope.cns.fr/brassicanapus/data/), and was visualized using the Circos software [35]. To identify gene duplication, the *GATA* genes were aligned using BLASTP with the e-value of 1e-10 and MCScanX to classify the duplication patterns including segmental and tandem duplication [36]. The tandem duplication was defined that a chromosomal region within 300 kb contains two or more genes [37]. Furthermore, the synteny relationships of *GATA* genes between the genomes of *B. napus* and *A. thaliana* were constructed according to Zhu et al. [38].

### Expression patterns of *BnGATA* genes in *B. napus*

To understand expression patterns of the *BnGATA* genes in *B. napus,* transcriptome data from 12 tissues of the *B. napus* cultivar “Zhongshuang 11” which was released in 2017 [25] were obtained from the NCBI (ID: PRJNA394926). We calculated and used the average expression level of three biological replicates of each tissue to show their expression patterns. Moreover, transcriptome data with three biological replicates of *B. napus* under dehydration, salt, ABA and cold stress conditions were obtained by referring to Zhang et al. [7, 9]. The fold changes (ratios to the control) of gene expression lower than 0.5 or higher than 2.0 were considered as differentially expressed genes (DEGs). These transcriptome data were available under the project ID: CRA001775 (https://bigd.big.ac.cn/). Expression standardization of *GATAs* was performed using the DSEeq2 R package and the heatmaps and the cluster analysis of *GATAs* were constructed using the TBtools software [39].

### SNP distribution of *GATAs* in a core collection of *B. napus*

To reveal natural variation of genomic sequences of *GATA* genes in *B. napus,* SNPs in the coding regions of *GATA* genes were determined in a worldwide collection of *B. napus* germplasm of 300 accessions in light of the genome re-sequencing data of our previous studies [26, 27]. High-quality SNPs with MAF larger than 5% and missing rate less than 50% were used for the further analysis.

## Results

### Identification and phylogenetic analysis of GATA proteins in *B. napus*

A total of 96 proteins with GATA zinc finger domain were identified to be the GATA family members in *B. napus* (Table S2). The longest sequence of each protein was remained, and the information of these proteins was listed in Table S2 and Table S3. The length of 96 GATA proteins was ranged from 101 to 576 amino acids (aa), and the molecular weight was ranged from 11.17 to 64.59 kDa.

**Fig. 1:**
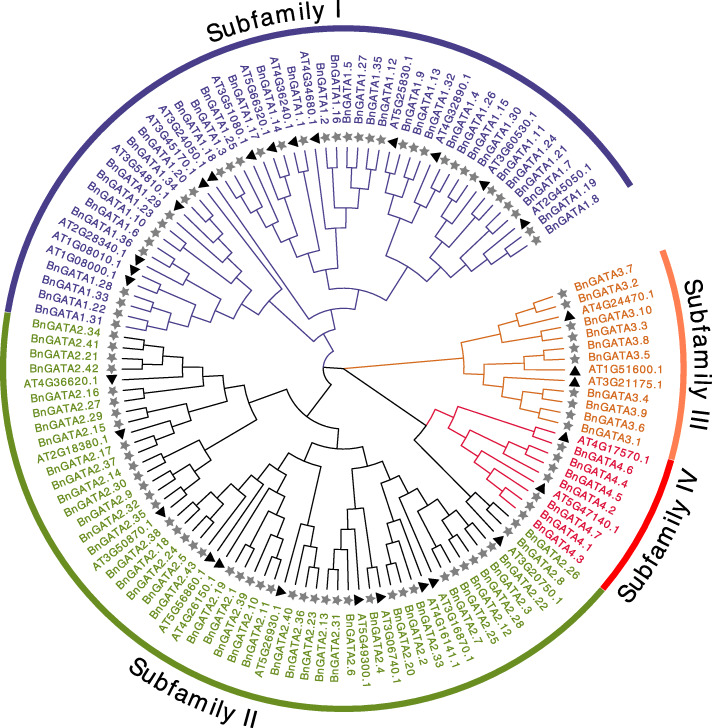
Phylogenetic analysis of GATA proteins in *B. napus* and *A. thaliana*. The different-colored arcs indicate subfamilies of the GATA proteins. The unrooted Neighbour-Joining phylogenetic tree was constructed using MEGA7 with full-length amino acid sequences of 126 GATA proteins, and the bootstrap test replicate was set as 1000 times. The asterisks and triangles represent the GATA proteins from *B. napus* and *A. thaliana*, respectively

To analyze the relationships of GATA proteins between *B. napus* and *A. thaliana*, an unrooted tree was constructed using the full-length amino acids of these GATAs. Totally, 30 proteins from *A. thaliana* and 96 proteins from *B .napus* were identified (Fig. 1). In *A. thaliana,* the GATAs were clustered into four subfamilies [2]. Here, 96 GATAs in *B. napus* were correspondingly classified into four subfamilies (Fig. 1). Among these GATA proteins, 36 members belong to the subfamily I, 43 to the subfamily II, 10 to subfamily III and 7 to the subfamily IV. Each BnGATA protein features with only one GATA domain. Notably, the GATA domain locates mainly in the position 160–230 aa for the subfamily I; 30–150 or 200–260 aa for the subfamily II; 190–330 aa for the subfamily III, and 7–40 aa for the subfamily IV, respectively (Table S2).

### Gene structures and protein motifs of *BnGATAs*

**Fig. 2:**
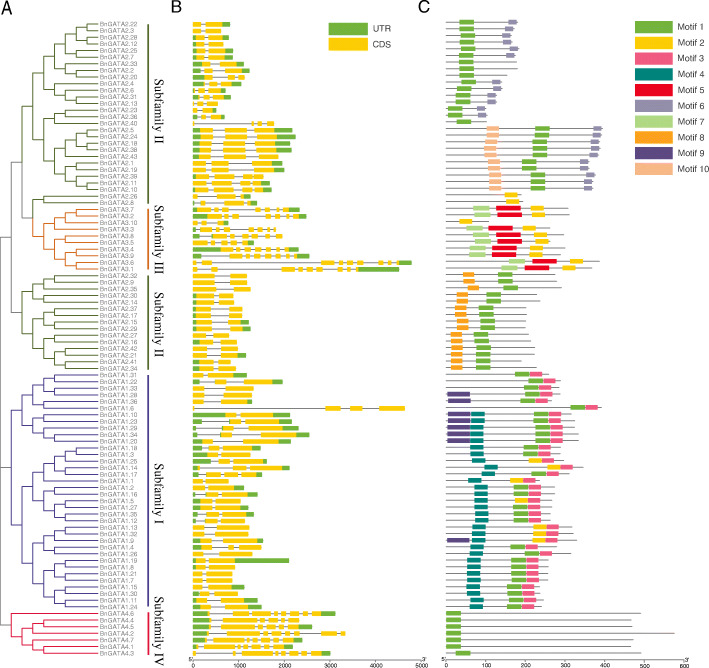
Schematic representation of phylogenetic relationships, gene structures and conserved motifs of the *GATA* genes in *B. napus.* **a** Phylogenetic tree of 96 BnGATA proteins. The unrooted neighbor-joining phylogenetic tree was constructed with MEGA7 using full-length amino acid sequences of 96 BnGATA proteins, and the bootstrap test replicate was set as 1000 times. **b** Exon/intron structures of *BnGATA* genes. Yellow boxes represent exons and black lines represent introns. The UTR region of *BnGATA* genes are indicated in green boxes. The length of exons can be inferred by the scale at the bottom. **c** The motif composition of BnGATA proteins. The motifs, numbers 1–10, are displayed in different colored boxes. The sequence information for each motif is provided in Table S4. The length of protein can be estimated using the scale at the bottom

As shown in Fig. 2b, one to nine exons were determined in *BnGATA* genes. Similar to *GATA* genes in *A. thaliana*, *BnGATA* genes in the subfamilies I and II have 2 to 3 exons except for *BnGATA1.6* (4 exons), 3 to 9 exons for the subfamily III, and 6 to 8 exons for the subfamily IV (Fig. 2b).

The motif analysis was conducted to display schematic structures of GATA proteins (Fig. 2c). The details of 10 kinds of conserved motifs were listed in Supplementary Table S4. The motif 1 and motif 2 were detected in all GATA proteins, the motif 3, 4 and 9 were mainly identified in the members of subfamily I, the motif 6, 8 and 10 were identified in the members of subfamily II, while the motif 5 and 7 were identified in the members of subfamily III. Except for the motif 1 and 2, no other motifs were found in the subfamily IV (Fig. 2c). In short, similar gene structures and conserved motifs within a subfamily strongly support the results of subfamily classifications by the phylogenetic analysis.

**Fig. 3:**
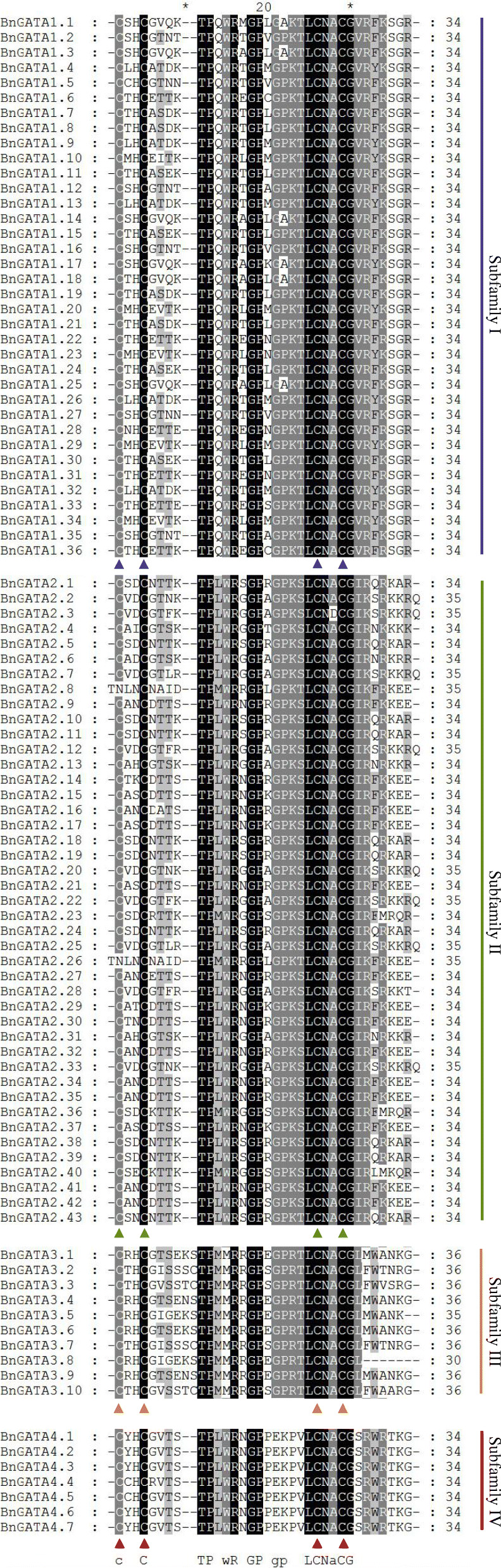
Alignments of GATA domain sequences of the GATA family members in *B. napus*. Highly conserved amino acid positions are marked with letters and triangles at the bottom

Moreover, with similar result of GATA domain analysis found in *A. thaliana* [2], BnGATAs in the subfamilies I, II and IV contained 18 residues in the zinc finger loop (C-X_2_-C-X_18_-C-X_2_-C), with the exception of BnGATA2.8 and BnGATA2.26, where N-X_2_-C-X_18_-C-X_2_-C appears instead of C-X_2_-C-X_18_-C-X_2_-C) (Fig. 3). All 10 BnGATAs in the subfamily III contained 20 residues between the second and the third Cys residues in the zinc finger (C-X_2_-C-X_20_-C-X_2_-C). In addition, several amino acid sites showed high conservation in the GATA domains such as LCNACG residues (Fig. 3).

### The distribution, genomic synteny and gene duplication of *BnGATA* genes

**Fig. 4:**
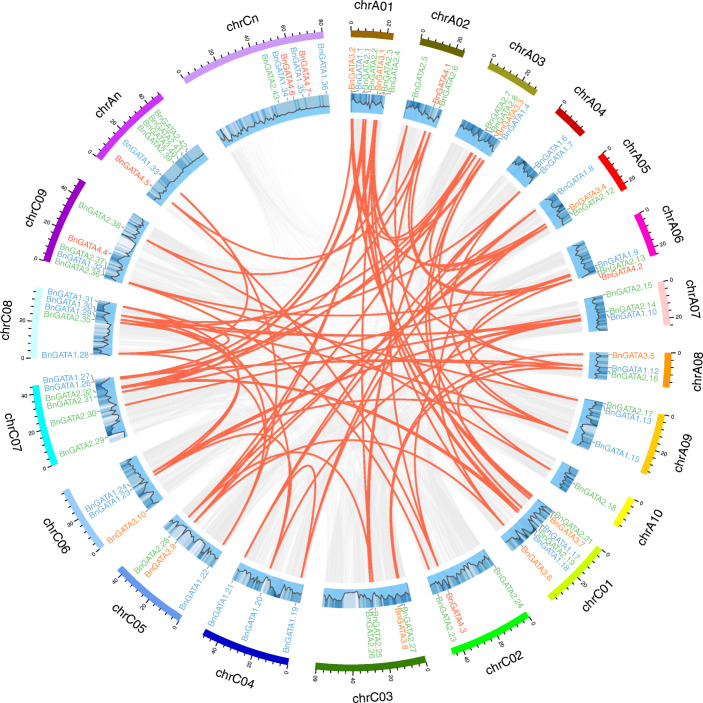
The chromosomal distribution and synteny analysis of *BnGATA* genes in *B. napus*. The locations of all the *BnGATA* genes are depicted in the chromosomes. Blue-colored genes belong to subfamily I, green-colored genes belong to subfamily II, orange-colored genes belong to subfamily III, red-colored genes belong to subfamily IV. Background gray lines indicate all *B. napus* genome synteny blocks, and the red lines highlight the duplicated *BnGATA* gene pairs. ID of the chromosomes is indicated at the bottom of each chromosome

Totally, 84 out of 96 *BnGATA* genes were distributed over 19 chromosomes, while other 12 genes were assigned into random fragments (6 on the AAnn subgenome and 6 on the CCnn subgenome) (Fig. 4 and Table S2). Among 84 *BnGATAs*, 46 genes located on the AA subgenome, including 16 subfamily I genes, 22 subfamily II genes, 5 subfamily III genes and 3 subfamily IV genes; while 50 genes located on the CC subgenome, including 20 subfamily I genes, 21 subfamily II genes, 5 subfamily III genes and 4 subfamily IV genes (Fig. 4). Some *BnGATA* genes were formed as clusters in the same chromosomes, such as *BnGATA1.32* and *BnGATA2.36* (Fig. 4). However, most *BnGATA* genes were randomly distributed on the AA or CC subgenome. In addition, Chr A1 showed the highest density of *BnGATA*s with 7 genes from the subfamilies II and III (Fig. 4).

Using BLAST and MCScanX methods, 82 segmental duplication events of the *GATA*s were identified (Fig. 4 and Table S5). Among these events, 80 duplication events occurred across chromosomes, while 2 events were detected within a chromosome (*BnGATA1.28/BnGATA1.31*, *BnGATA1.19/BnGATA1.21*). Furthermore, 14 duplication events took place on the AA subgenome, 14 events on the CC subgenome, and 50 events across AA/CC subgenomes. The results suggest that some *BnGATA* genes possibly came into being during gene duplication, and the segmental duplication events could play key roles in the expansion of *BnGATA* genes in *B. napus*.

**Fig. 5:**
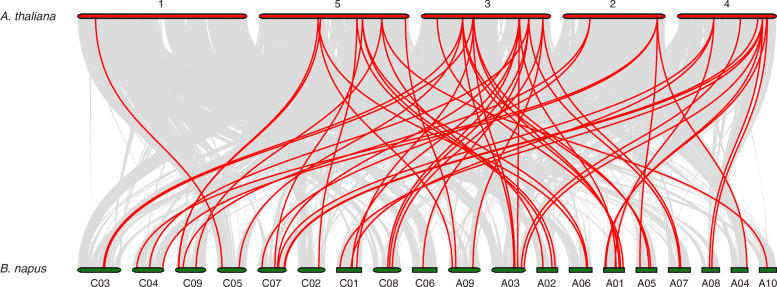
Synteny analysis of *GATA* genes between *B .napus* and *A. thaliana*. Gray lines indicate all collinear blocks within *B. napus* and *A. thaliana*, while the red lines depict the orthologous relationships of *GATA* genes between *B. napus* and *A. thaliana*

To better understand the evolution of *BnGATA* genes, the synteny of the *GATA* gene pairs between the genomes of *B. napus* and *A. thaliana* was constructed (Fig. 5 and Table S6). Here, 55 *BnGATAs* exhibited syntenic relationship with *AtGATAs*. Some *AtGATAs* were associated with more than one orthologous copies in *B. napus.* For example, *AT2G45050* showed syntenic relationship with *BnGATA1.7, BnGATA1.8, BnGATA1.19* and *BnGATA1.21* (Table S6). Moreover, collinear gene pairs of *GATA* genes fixed on highly conserved syntenic blocks were also detected (Fig. 5 and Table S6).

### Expression profiles of *BnGATAs* in different tissues

**Fig. 6:**
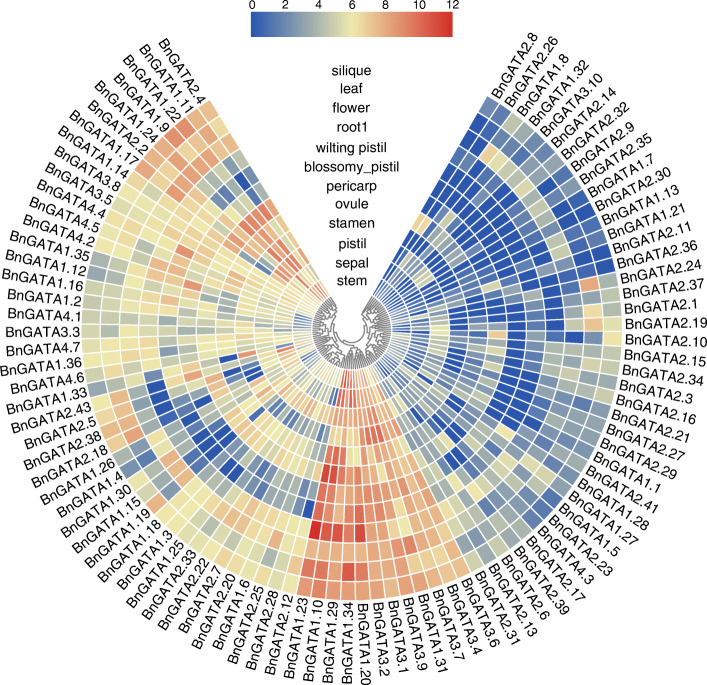
Expression profiles of *BnGATA* genes in different tissues. Expression data were processed with log_2_ normalization. The color scale represents relative expression levels from high (red colored) to low (blue color)

The expression profiles of 96 *BnGATA* genes in 12 tissues of the rapeseed cultivar ZS11 were compared (Fig. 6 and Table S7). According to the difference of their expression pattern, these genes were clustered into three groups. In details, a total of 39 genes were classified into the group 1 showing low expression levels or not detected in the tissues examined. 12 *BnGATAs* were belonging to the group 2 with high expression levels in these tissues. Meanwhile, 43 *BnGATAs* were included in the group 3 showing preferential expression profiles across tissues. For instance, *BnGATA1.11* was not expressed in wilting pistil, expressed with low levels in blossomy pistil and root, but expressed highly in other tissues (Fig. 6 and Table S7).

On the other hand, the group 1 contained 9, 28, 1 and 1 genes from the four subfamilies; the group 2 had 6 and 6 genes from the subfamilies I and III, while the group 3 contained 21, 13, 3 and 6 genes from the four subfamilies, respectively (Table S7). Interestingly, it was found that *BnGATAs* from the subfamily II showed low expression levels in all tissues, but the subfamily III members had high expression levels in all tissues (Fig. 6 and Table S7). The expression patterns of *GATA* genes in different tissues suggested functional divergences between different subfamilies.

### Expression profiles of *BnGATAs* in response to abiotic stresses

**Fig. 7:**
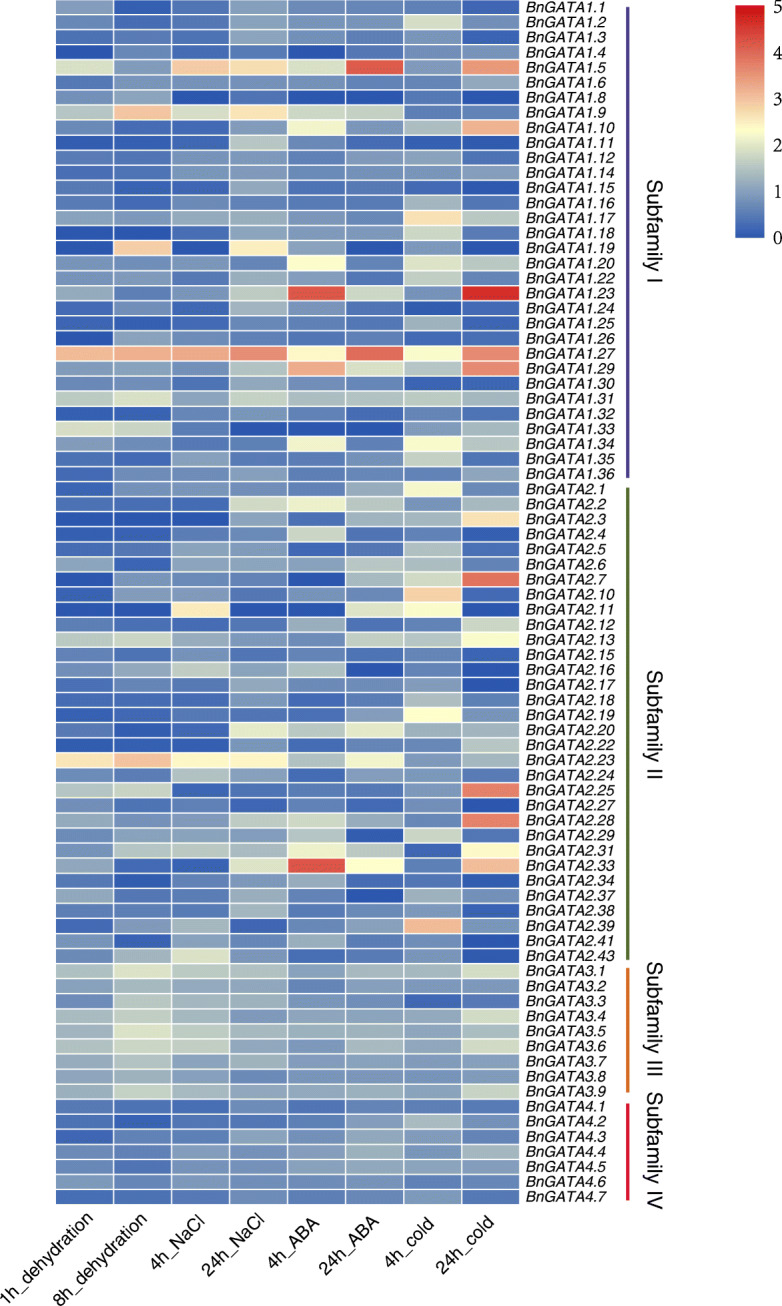
Expression profiles of *BnGATA* genes under abiotic stress condition. Expression profiles were shown as the ratios of the values of the treatments to the controls and processed with log_2_ normalization. The color scale represents relative expression levels from high (red colored) to low (blue color)

Further, we studied the expression pattern of *BnGATA* genes under various abiotic stresses including drought, salinity, ABA induction and cold stresses (Fig. 7 and Table S8). In detail, most genes of the subfamily III members were remarkably up-regulated, while most of the subfamily IV genes were down-regulated in response to dehydration and salt treatments. *BnGATA1.27, BnGATA2.23* and *BnGATA3.1* were up-regulated, but *BnGATA1.8* was not expressed after salt treatment. Under dehydration stress, *BnGATA1.9, BnGATA1.27* and *BnGATA2.23* showed the largest increase in expression levels*,* while *BnGATA1.11* and *BnGATA2.5* were significantly decreased. *BnGATA1.27* and *BnGATA2.33* showed higher expression level under ABA induction, while *BnGATA2.5* was down-expressed. Under cold stress, *BnGATA1.23* and *BnGATA1.29* were significantly up-regulated, while *BnGATA1.11* and *BnGATA1.24* were remarkably down-regulated. Notably, *BnGATA1.27* was significantly induced by all abiotic stresses (Fig. 7). Besides, *BnGATA1.9, BnGATA1.29* and *BnGATA2.5* could respond to diverse abiotic treatments (Fig. 7, Table S8). The results of the expression profiles of *BnGATA* genes under diverse abiotic treatments may suggest their functional differences among four subfamilies.

### Sequence variation of *BnGATAs* in a core collection of *B. napus*

Based on our previous re-sequencing data of 991 worldwide accessions of rapeseed [26, 27], the SNPs from 300 core accessions with MAF more than 5% were used for the analysis. In average, 6 SNPs were detected for a *GATA* gene (Table S9). It was found that the SNP density of *BnGATA*s on the AA subgenome was higher than that on the CC subgenome (Table S9). Meanwhile, the SNP density of each subfamily was different, with averagely 6.7, 3.58, 14.2 and 7.14 SNPs for the four subfamilies, respectively.

**Fig. 8:**
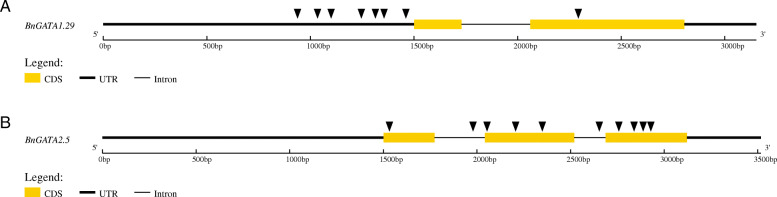
Gene structures and SNPs location of *BnGATA1.29* and *BnGATA2.5*. 8 and 10 SNPs were identified in *BnGATA1.29* (**a**) and *BnGATA2.5* (**b**) respectively. SNPs location is indicated with black triangle

The SNP density of each *BnGATA* gene within a subfamily was also different. For instance, no SNP was identified for *BnGATA1.27*, while 8 and 10 SNPs were identified for *BnGATA1.29* and *BnGATA2.5*. Moreover, a detailed SNP distribution of *BnGATA1.29* and *BnGATA2.5* were shown in Fig. 8. For *BnGATA1.29,* it was found that there were 6 SNP loci in the promoter region, 2 SNPs in the exon/intron region and no SNP in the 3’UTR region (Fig. 8a)*.* For *BnGATA2.5,* there were no SNP in the promoter region, 10 SNPs in the exon/intron region and no SNP in the 3’UTR region (Fig. 8b). We speculate that sequence variation of these *GATA*s may be related to their expression difference under abiotic stresses.

## Discussion

In this study, we identified 96 genes of *GATA* family transcription factors in *B. napus*, designating as *BnGATA1.1* to *BnGATA4.7* based on their subfamily classification. Bioinformatics analyses such as phylogenetic relationships, domains, gene structures, protein motifs, chromosomal locations, homologous and orthologous genes of *GATA* were performed. The results indicate that *BnGATAs* clustered into four subfamilies are significantly different with genetic structures and expression patterns, and which are more complex than the *GATA* TFs in *A. thaliana*. Furthermore, the information on gene transcription level and SNP distribution provides a resource for functional identification of *BnGATAs*. The results provide a valuable resource for functional identification of *BnGATA* TFs and molecular breeding in *B. napus*.

In previous studies, the *GATA* family genes were systematically investigated in *A. thaliana* and *O. sativa* [2, 40], *Solanum lycopersicum* [5], *Vitis vinifera* [8], *Phyllostachys edulis* [6] and *Gossypium genues* [7, 9]. According to these studies, the *GATA* genes from dicotyledons, but not from monocots, could be strictly divided into four subfamilies. In our study, we also find that the subfamilies I, II and III of the *GATA* genes simultaneously occur in both dicotyledons and monocots, but the subfamily IV genes did not exist in monocots [2, 6]. It demonstrated that the subfamily IV of *GATA* genes appeared after the divergence between dicotyledon and monocot. Therefore, we speculate that the *GATA* subfamily IV genes may play unique functions in dicotyledonous plants, but further evidence is needed.

Significant differences in gene and protein structures among *BnGATA* subfamilies may lead to functional divergences. For example, in subfamily III, the GATA domain featured with 20 residues in the zinc finger (C-X_2_-C-X_20_-C-X_2_-C), while there were 18 residues in the other three subfamilies. The CCT and TIFY domains were specifically found in the subfamily III, which were reported to be involved in flowering, hypocotyl and root development in *A. thaliana* [41–43]. The subfamily I genes may be involved in plant growth and respond to abiotic stresses. In *A. thaliana, BME3* (ortholog of *BnGATA1.29*) was reported as a positive regulator for seed germination [44]. The *BME3* knockout plants showed deeper dormancy and more sensitive to cold stress than the wild-type plants. Moreover, the decreased expression of *GA20-oxidase* and *GA3-oxidase* in the knockout plants suggested that *BME3* was involved in GA biosynthesis [44]. In this study, *BnGATA1.29* (*BnaC08g25560D*) exhibited high expression levels in various tissues and significantly responded to ABA and cold stresses (Table S7 and Table S8). A recent study reported that *RGL2-DOF6* complex regulates *GATA12* (from the subfamily I) gene to enforce primary dormancy in *A. thaliana* [45]. The subfamily II of *BnGATAs* is involved in plant flowering and abiotic stress responding. In *A. thaliana, GNC* and *GNL* (ortholog of *BnGATA2.5*) were involved in germination, greening, flowering, floral development, senescence and floral organ abscission [10–12, 46–49]. Recently, the association between *BnGATA2.5* gene expression and plant height, branch initiation height and flowering time was detected in *B. napus* [50]. In this study, *BnGATA2.5* (*BnaA02g08490D*) was expressed across many tissues and organs in *B. napus* (Fig. 6, Table S7). Moreover, the expression of *BnGATA2.5* was down-regulated under ABA inducement, drought and cold treatments, indicating its strong response to abiotic stresses (Fig. 7, Table S8). The subfamily III of *GATA* TFs is a novel plant-specific subfamily, which plays important roles in flowering, hypocotyl and root development [41–43]. For instance, overexpression of *ZIM* (*GATA25*) could up-regulate the expression of *XTH33* (*xyloglucosyl transferase 33*), resulting in elongate hypocotyls and prtioles in *A. thaliana* [42, 43]. Besides, *ZML1* (*GATA24*) and *ZML2* (*GATA28*) were identified as the two essential components of the *cry1* (Cryptochrome1)-mediated photoprotective response in *A. thaliana* [51]. In this study, *BnGATA3.1* (*BnaA01g25320D*) as the ortholog of *AtZML1*, was highly expressed in most tissues in *B. napus* (Fig. 6, Table S7). The expression of *BnGATA3.1* was slightly changed in response to a variety of abiotic stresses (Fig. 7, Table S8). However, so far, little was known about the subfamily IV of the *GATA* TFs in plants.

In this study, we found that *BnGATA* genes had a plentiful genetic variation of SNPs in a core collection of *B. napus*. SNPs in the coding regions are crucial for the generation of new alleles, and allele divergence may lead to gene function alterations, which is vital facilitation for crop species adaptation to environmental stresses [52]. For example, 7 functional alleles of powdery mildew resistance gene *Pm3* were isolated from a set of 1320 bread wheat landraces through allele mining, while the other 9 alleles of *Pm3* showed non-function to powdery mildew resistance [53]. In our core collections of rapeseed, the SNP density of the subfamily III genes (5.7 SNPs per 1 kb) was averagely higher than that in the other subfamily genes (3.5) (Table S9), while the subfamily III genes were highly expressed in various tissues and under dehydration condition (Table S7 and Table S8). Therefore, haplotypes and allele-specific markers of *BnGATA* genes could be identified for rapeseed molecular-breeding programs in future works. Rapeseed originated from the natural crossing between *B. rapa* (AA) and *B. oleracea* (CC) [24]. In this study, we identified 46 and 50 *BnGATA* genes located on the AA or CC subgenomes. However, the SNP density of *BnGATAs* on the AA subgenome (4.7 per 1 kb) was much higher than that on the CC subgenome (3.2) (Table S9), which could be explained by more frequent outcrossing between *B. napus* and *B. rapa* than between *B. napus* and *B. oleracea* [26].

Taken together, we performed a comprehensive characterization of *GATA* family genes in *B. napus*. The results enrich our knowledge about *BnGATA* genes, providing a basis for manipulation of the genes and facilitating breeding marker-assisted breeding in rapeseed. However, functional validation is needed to reveal the exact functional roles of *BnGATA* genes.

## Conclusion

In the present study, genome-wide identification and characterization of *GATA* genes were conducted in *B. napus.* A total of 96 *GATA* genes are identified in the rapeseed genome, which were divided into four subfamilies. Phylogenetic and synteny analysis of *GATA* genes between *A. thaliana* and *B. napus* provide valuable clues for the evolutionary characteristics of the *BnGATA* genes. Moreover, gene expression and SNP distribution analysis of *BnGATA* genes were also determined. These results provide insights into the functional differences, evolutionary relationships and expression profiles of *GATA* transcription factors in *B. napus*.

## Supplementary Information

**Additional file 1: Table S1.** The information of the *GATA* genes in *A. thaliana*. **Table S2.** Characteristics of the *GATA* genes in *B. napus*. **Table S3.** The list of 96 *GATA* genes identified in *B. napus*. **Table S4.** Conserved amino acid motifs and annotation of the *GATA* genes in *B. napus*. **Table S5.** Syntenic blocks of the *GATA* genes in *B. napus*. **Table S6.** One-to-one orthologous relationships of the *GATA* genes between *B. napus* and *A. thaliana*. **Table S7.** The expression profiles (log2-based values) of the *GATA* genes in different tissues of *B. napus*. **Table S8.** The expression profiles (ratio to control values) of the *GATA* genes in *B. napus* under abiotic stresses. **Table S9.** SNPs of the *GATA* genes identified in 300 core collections of rapeseed germplasm. **Table S10.** Genetic diversity of 300 core collections of rapeseed germplasm.

## Data Availability

RNA-seq of *B. napus* variety Zhongshuang 11 (ZS11) in distinct tissues are available in the NCBI Sequence Read Archive (SRA) database under the accession number PRJNA394926. RNA-seq data of *B. napus* under dehydration, salt, ABA and cold stress conditions were available under the project ID: CRA001775 (https://bigd.big.ac.cn/). All other datasets supporting the results of this article are included within the article and its supplementary tables.

## Abbreviations

*ARF*: Auxin response factors
*AtGATA*: *Arabidopsis thaliana GATA*
BLASTP: Basic local alignment search tool-protein
*BnGATA*: *Brassica napus GATA*
*bZIP*: Basic helix loop helix
*cry1*: Cryptochrome1
DREB: Dehydration-responsive element-binding protein
GA: Gibberellin
*GNC*: GATA, nitrate-inducible, carbon-metabolism involved
*GNL*: GNC-like
GSDS: Gene structure display server
HMM: Hidden markov mode
MEME: Motif elicitation
MW: Molecular weights
*MYB*: V-myb avian myeloblastosis viral oncogene homolog
pI: Isoelectric point
*PIFs*: Phytochrome interacting factors
SNP: Single nucleotide polymorphisms
*SOC1*: Suppressor of constans 1
TFs: Transcription factors
*XTH33*: Xyloglucosyl transferase 33
